# *In Vivo* and *In Vitro* Study on the Efficacy of Terpinen-4-ol in Dextran Sulfate Sodium-Induced Mice Experimental Colitis

**DOI:** 10.3389/fimmu.2017.00558

**Published:** 2017-05-12

**Authors:** Zecai Zhang, Peng Shen, Xiaojie Lu, Yanxin Li, Jiuxi Liu, Bo Liu, Yunhe Fu, Yongguo Cao, Naisheng Zhang

**Affiliations:** ^1^College of Veterinary Medicine, Jilin University, Changchun, China

**Keywords:** terpinen-4-ol, colitis, NLRP3 inflammasome, interleukin-1β, intestinal microbiota, intestinal barrier, lipopolysaccharide

## Abstract

The purpose of this study was to investigate the protective effects of Terpinen-4-ol (TER) on dextran sulfate sodium (DSS)-induced experimental colitis and clarify the possible mechanisms. *In vivo*, an acute colitis model was used to confirm the anti-inflammatory activity and the possible mechanisms of TER in C57BL/6 and NLRP3^−/−^ mice. *In vitro*, we performed further study, using RAW264.7 cells and Caco-2 cells, to confirm the molecular mechanisms of TER on inflammatory response. In C57BL/6 mice, TER alleviated DSS-induced disease activity index (DAI), colon length shortening, colonic pathological damage, and myeloperoxidase (MPO) activities. The production of pro-inflammatory mediators was significantly decreased by TER. Furthermore, TER inhibited NF-κB and NLRP3 inflammasome activation. Surprisingly, TER reduced the plasmatic lipopolysaccharide (LPS) concentration and re-balanced *Escherichia coli* (*E. coli*) and *Lactobacillus* levels. In addition, TER prevented the impairment of colon epithelium barrier by regulating the expression of zonula occludens-1 and occludin. *In vitro*, the results showed that TER significantly suppressed NLRP3 inflammasome activation in LPS-stimulated RAW264.7 cells, as indicated by decreased expression of NLRP3 and caspase-1, and lowered interleukin-1β secretion. In contrast, mice deficient for NLRP3 were less sensitive to DSS-induced acute colitis, and TER treatment exerted little protective effect on DSS-induced intestinal inflammation in NLRP3^−/−^ mice. The protective effect of TER may be largely attributed to its inhibition of NLRP3 inflammasome activation in colon. Taken together, our findings showed that TER might be a potential agent for the treatment of ulcerative colitis.

## Introduction

Inflammatory bowel diseases (IBD), such as Crohn’s disease and ulcerative colitis (UC), is a non-specific, chronic, and relapsing inflammation of the gastrointestinal (1). UC, an intractable IBD, is characterized by weight loss, diarrhea, rectal bleeding, and abdominal pain, which not only affects millions of patients worldwide but also increases the risk of colon cancer (2, 3). Although the etiology and pathogenesis of UC are complicated and remain uncertain, genetic susceptibility of the host, the host immune system, the intestinal microflora, and changed colonic barrier function have been found to be about the developments and course of UC (4).

Nuclear factor-κB (NF-κB) has represented a paradigm for signal transduction and gene regulation related with numerous diseases. Once activated, NF-κB induces the production of pro-inflammatory cytokines, such as tumor necrosis factor-α (TNF-α), interleukin-1β (IL-1β), and interleukin-12 (IL-12) (5). In addition, emerging evidence suggests the pivotal role of NOD-like receptor family pyrin domain containing 3 (NLRP3) in the developments and pathogenesis of IBD (6). The NLRP3 activation could lead to the maturation and secretion of highly pro-inflammatory IL-1β.

The immune system is pivotal in regulating the interactions between the host and the intestinal microflora, while the composition of the bacterial flora in the gut strongly impacts the outcome of the immune response (7). Several studies have also indicated that IBD is affected by the disturbance in the composition of gut microbiota (8, 9). The disturbance of intestinal microflora could increase lipopolysaccharide (LPS)-producing bacterial growth and LPS production in the intestine which increases the LPS absorption from the gastrointestinal tract into the blood and activates inflammatory signaling pathways continuously, leading to chronic inflammatory diseases, such as colitis (10, 11). The epithelial barrier acts as the first line of defense against varieties of harmful substances, which results in decreased intestinal permeability and the inhibition of inflammation. It has been reported that the expression of colonic tight junction (TJ) proteins regulates the LPS absorption from the gastrointestinal tract into the blood (12). Therefore, the repair of TJ proteins expression could decrease the absorption of LPS and reduce inflammation (13).

Dextran sulfate sodium (DSS) is a chemical which is wildly used to induce colitis (14). Colitis induced by DSS represents a well-established model which could research into the pathogenesis of UC and is similar to human UC (15). In the past decades, most therapeutic drugs for UC include salicylazosulfapyridine (SASP), immunosuppressive agents, and anti-TNF-α monoclonal antibody. Unfortunately, most of these agents have severe side effects or high cost particularly for long-term therapy (16). Therefore, it is necessary to develop therapeutic strategies. Terpinen-4-ol (TER) is a kind of main components of essential oil from *Zanthoxylum bungeanum* Maxim. TER, which demonstrated antioxidant effects together with anti-inflammatory properties in LPS-stimulated human monocytes (17, 18), showed high antibacterial activity toward *Salmonella typhimurium, Staphylococcus aureus*, and *E. coli in vitro* (19). However, direct evidence for the effect of TER on mice colitis has not yet been elucidated. Here, we examined the protective effects and clarified the possible mechanisms of TER *in vivo* and *in vitro*.

## Materials and Methods

### Materials

Dextran sulfate sodium (molecular weight of 36–50 kDa) was obtained from MP Biomedicals (Irvine, CA, USA). TER was obtained from TCI Chemical Industry Co., Ltd. (Shanghai, China). Rabbit mAb, IκBα, and p65 and mouse p-IκBα monoclonal antibodies and p-p65, NLRP3, caspase-1, ASC, and IL-1β were provided by Cell Signaling Technology Inc. (Beverly, MA, USA). The primary antibodies that were raised against occludin and zonula occludens-1 (ZO-1) were obtained from Santa Cruz (Santa Cruz, CA, USA). β-actin and horseradish peroxidase conjugated goat anti-rabbit and goat anti-mouse antibodies were purchased from Tianjin Sungene Biotech Co., Ltd. (Tianjin, China). The myeloperoxidase (MPO) determination kit was purchased from the Jiancheng Bioengineering Institute of Nanjing (Nanjing, China). All enzyme-linked immunosorbent assay (ELISA) kits were obtained from Biolegend (San Diego, CA, USA). The Nuclear and Cytoplasmic Protein Extraction Kit was provided by Beyotime Institute of Biotechnology (Jiangsu, China). All other chemicals were of reagent grade.

### Animals

Male C57BL/6 mice (21–23 g) were provided from the Center of Experimental Animals of Jilin University, China. NLRP3^−/−^ mice were on C57BL/6 background. The mice were housed in an air-conditioned room with the laboratory temperature maintained at 24 ± 1°C and provided water and food *ad libitum*. All experimental protocols were guided in accordance with the approval of the Institutional Animal Care and Use Committee of our university under the approved protocol number SCXXK (JI-2016-0003).

### Establishment of DSS-Induced Mice Colitis Model and Treatment

**Figure 1:**
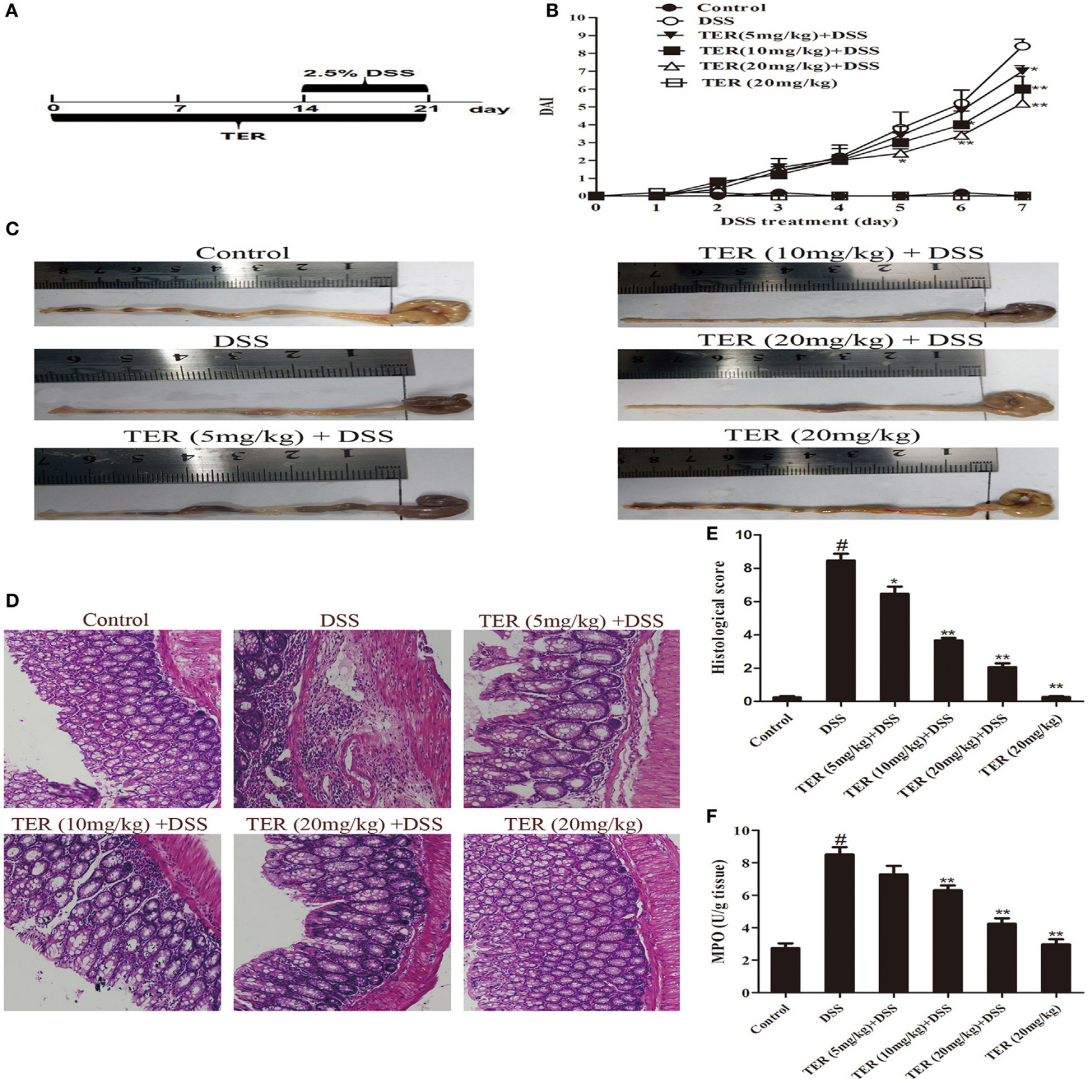
**Terpinen-4-ol (TER) attenuates dextran sulfate sodium (DSS)-induced colitis in C57BL/6 mice**. Mice were treated with 2.5% DSS in their drinking water for 7 days to induce acute colitis. TER (5, 10, 20 mg/kg) was administered for 14 days before and during DSS treatment *via* oral gavage once per day. Mice were sacrificed at day 21 after colitis induction. **(A)** The experimental protocol with TER in acute colitis model. **(B)** Disease activity index (DAI) during the disease process. **(C)** The lengths of colons from each group of mice were measured. **(D)** The colons from each experimental group were processed for histological evaluation (hematoxylin–eosin staining, 200×; scale bar, 50 µm). **(E)** Histopathological scores of each group were determined. **(F)** Myeloperoxidase (MPO) activity in the colonic tissues was detected. The results are representative of at least three independent experiments and expressed as mean ± SD. *p < 0.05 and **p < 0.01 vs the DSS-treated group on the same day; ^#^*p* < 0.05 vs the control group.

Acute colitis was induced by feeding mice with 2.5% (w/v) DSS, which was dissolved in drinking water, continuously for 7 days. The experiment was randomly divided into six groups: control group;DSS group; TER (5, 10, 20 mg/kg) + DSS groups, and TER (20 mg/kg) group. The experimental time lines of the animal model are described in Figure 1A. To assess chemoprevention effect of TER on DSS-induced acute colitis in C57BL/6 mice, the mice were treated with indicated dose of TER 14 days before DSS treatment once per day. Subsequently, to further prove the key role of NLRP3 in the effect of TER, TER was administered intragastrically daily at 20 mg/kg. Wild-type (WT) or NLRP3^−/−^ mice were randomly assigned to control group, DSS group, and TER (20 mg/kg) + DSS group. The experimental time lines of the animal model are described in Figure 1A.

### Clinical Scoring and Histological Analysis

In all groups, mice weight, stool characters, and blood in stool were recorded daily. The disease activity index (DAI) evaluations were determined by previously established scoring system (20). Briefly, DAI scores were based on the following: changes in body weight, consistency of stool, and hemoccult bleeding. These parameters were assessed on a scale from 0 to 4, with 4 being the most severe. The DAI data are presented as an average score of these parameters taken daily. Animals were sacrificed by cervical dislocation under anesthesia, and the colon was excised from cecum to 1 cm above the anus. The length of colon was detected. For histological analysis, the colon specimens were fixed in 10% formalin and embedded in paraffin. Sections were stained with hematoxylin–eosin (H&E) according to standard protocols. Histological scoring was performed using a previously described method (21).

### MPO Assay

Myeloperoxidase activity reflects the number and distribution of neutrophils in the tissues. The colon tissues were weighed and homogenized with phosphate-buffered saline (PBS) [1:9 (w/v)]. The supernatants were collected. The activity of MPO was measured according to the manufacturer’s instructions.

### Cell Culture and Viability Assay

The RAW264.7 cells were purchased from China Cell Line Bank (Beijing, China) and cultured in RPMI-1640 medium with 10% fetal bovine serum (FBS; HyClone, Logan, UT, USA), 100 U/mL penicillin, and 100 U/mL streptomycin at 37°C with 5% CO_2_.

Human intestinal epithelial cells (Caco-2 cells) were cultured with 15% FBS/DMEM-F12 supplied with 1 mM sodium pyruvate and 50 U/mL penicillin–streptomycin. Caco-2 cells were maintained at 37°C in a humidified 5% CO_2_ incubator.

Cells were pretreated with or without TER (0.025, 0.05, and 0.1 mM) for 1 h. After that, cells were treated with LPS (1 µg/mL). After 18 h of LPS stimulation, MTT (20 µL of 5 mg/mL) was added to each well for 4 h. The supernatant was removed and dimethyl sulfoxide (150 µL per well) was added. The optical density was tested at 570 nm using a microplate reader.

### Preparation of Cecal Bacterial Lysates

Cecal bacterial lysates (CBLs) were prepared as described by Dieleman et al. (22). Briefly, the cecal contents in each group were solubilized by vortexing the contents in RPMI-1640 medium and then incubating them with 0.01 M MgCl_2_, 10 µg/mL DNase. Then, the contents were homogenized with 0.1-mm glass beads for 3 min. The homogenate was centrifuged at 10,000 × *g* for 10 min. The supernatant was filtered through a 0.45-µm syringe filter.

### Mesenteric Lymph Node Cell Cultures

Mesenteric lymph node (MLN) was harvested from mice of six experimental groups. Single-cell suspensions were prepared as described by Ruyssers et al. (23). Briefly, approximately 4 × 10^5^ MLN cells and 20 µg/mL CBL were cultured in RPMI-1640 medium with 10% FBS and 50 mg/mL gentamicin at 37°C with 5% CO_2_ for 72 h. The culture media was then collected for cytokine analysis and stored at −20°C.

### Cytokine Assays

Adipose tissue was removed from excised colons and washed with PBS. Colon tissues (about 50 mg per well) were cultured in RPMI-1640 medium (100 mg/mL of streptomycin and 100 U/mL of penicillin) at 37°C in 5% CO_2_. After 24 h, colon supernatants were centrifuged at 12,000 × *g* at 4°C for 10 min. The RAW264.7 cells were pretreated with or without TER (0.025, 0.05, and 0.1 mM) for 1 h, and then stimulated with 1 µg/mL of LPS for 18 h. The TNF-α, IL-1β, and IL-12 autocrine levels in the colon were detected with ELISA kits according to the manufacturer’s protocol.

### Quantitative Real-time Polymerase Chain Reaction

Total RNA was extracted from mouse colon samples and cells using the TRIzol reagent as per the manufacturer’s protocol (Invitrogen). The RNA was reverse-transcribed into complementary DNA (cDNA) using a Revert Aid First Strand cDNA Synthesis Kit (Thermo Scientific). Quantitative real-time PCR (qRT-PCR) was performed with a 7500 Fast Real-Time PCR System (Applied Biosystems) and the SYBR Green Plus reagent kit (Roche), as described elsewhere (24). The sequences of primers were listed in Table 1. β-actin was used as the reference gene.

**Table 1:** **Oligonucleotide primers used for quantitative real-time PCR**.

Name	Primer sequence
Tumor necrosis factor-α	Sense: 5′-GCCTCCCTCTCATCAGTTCTA-3′
	Anti-sense: 5′-GGCAGCCTTGTCCCTTG-3′
Interleukin-1β	Sense: 5′-ACCTGTGTCTTTCCCGTGG-3′
	Anti-sense: 5′-TCATCTCGGAGCCTGTAGTG-3′
Interleukin-12	Sense: 5′-GGTCACACTGGACCAAAGGGACTATG-3′
	Anti-sense: 5′-ATTCTGCTGCCGTGCTTCCAAC-3′
Zonula occludens-1	Sense: 5′-GTTCCGGGGAAGTTACGTGC-3′
	Anti-sense:5′-AAGTGGGACAAAAGTCCGGG-3′
Occludin	Sense: 5′-GTTCCGGGGAAGTTACGTGC-3′
	Anti-sense: 5′-AAGTGGGACAAAAGTCCGGG-3′
β-actin	Sense: 5′-CTACCGTCGTGACTTCGC-3′
	Anti-sense: 5′-GGGTGACATCTCCCTGTT-3′
*Escherichia coli*	Sense: 5′-GCGTTGCGTAAATATGGTTGCCG-3′
	Anti-sense: 5′-CGTCACAGGCTTCAATCATGCGTT-3′
*Lactobacillus*	Sense: 5′-CACCGCTACACATGGAG-3′
	Anti-sense: 5′-AGCAGTAGGGAATCTTCCA-3′

### Western Blotting Analysis

Colon samples were homogenized. Total protein was extracted as per the manufacturer’s protocol. Cells were treated with TER (0.025, 0.05, and 0.1 mM) for 1 h. After LPS treatment for 1 h, total cellular protein was extracted. Protein contents were quantified and transferred onto PVDF membrane. Next, the blocked membranes with 5% non-fat milk were incubated overnight at 4°C with primary antibodies against p65 (1:1,000), p-p65 (1:1,000), IκBα (1:1,000), p-IκBα (1:1,000), NLRP3 (1:500), caspase-1 (1:1,000), ASC (1:250), IL-1β (1:1,000), ZO-1 (1:300), occludin (1:300), and β-actin (1:1,000). The secondary antibodies used were goat anti-rabbit or -mouse IgG (1:20,000) and were incubated with membranes at room temperature for 2 h. The blots were tested using a western blotting detection program. β-actin was used as an internal control.

### Immunohistochemistry (IHC)

Immunohistochemical stains against ZO-1 and occludin were detected using IHC kit (MaiXin, China). Briefly, paraffin-embedded slides were deparaffinized, rehydrated, and washed in 1% PBS. After that, they were incubated with 3% hydrogen peroxide and blocked with 10% goat serum for 1 h at 37°C. Then, slides were treated with primary antibodies (1:100) overnight at 4°C. Biotinylated secondary anti-rabbit antibodies were added and incubated at room temperature for 1 h. Streptavidin-HRP was added, and after 40 min the sections were achieved using diaminobenzidine as a chromogen and counter-stained with hematoxylin. Images at 200× magnification were examined with a microscope (Olympus, Japan).

### Immunofluorescence (IF)

Immunofluorescence was performed on paraffin-embedded colonic tissue sections. The sections were deparaffinized, rehydrated, and washed in 1% PBS-Tween. After that, they were treated with 3% hydrogen peroxide, blocked with 10% goat serum, and incubated with ZO-1 and occludin primary antibody in PBS-Tween containing 1% BSA (1:50) for 1 h at 37°C. Slides were washed and incubated for 1 h with species-specific fluorescently labeled secondary antibodies. The slides were stained with DAPI. Cover slips were mounted and analyzed on a confocal laser microscope.

### Determination of LPS

Plasma endotoxin contents were detected by Limulus amebocyte lysate (LAL) assay kit according to manufacturer’s protocol. Briefly, plasma (5 µL) was diluted 1:10 in pyrogen-free water, inactivated at 70°C for 10 min, and incubated with LAL solution at 37°C for 30 min. Addition of reagents led to formation of a magenta derivative that absorbs light at 545 nm.

### Bacteriologic Analysis of Intestinal Bacteria

The mice were previously marked so individual mice could be followed in the whole experiment. In order to collect stool samples, individual mice were put in isolation containers. For bacterial community analysis, stool samples were weighed and stored at −80°C. DNA was extracted using a phenol–chloroform extraction technique with mechanical disruption (bead-beating) based on a previously described protocol (25). For the preparation of a standard for *Escherichia coli* (*E. coli*) and *Lactobacillus*, a clone obtained from mice was used. The sequences of specific bacterial primers were listed in Table 1. Results are expressed as 16SrRNA gene copies per wet weight of feces.

### Statistical Analysis

All data are shown as the mean ± SD. One-way analysis of variance (Dunnett’s test) was used where three or more groups of data were compared. All experiments were repeated at least three times. *p* ≤ 0.05 were considered statistically significant.

## Results

### TER Attenuates DSS-Induced Colitis in C57BL/6 Mice

To investigate the protective effect of TER on UC, we established a model of DSS-induced mice colitis by feeding C57BL/6 mice with drinking water containing 2.5% DSS for 7 days. The mice treated with only DSS had a significant increase in DAI scores compared with control group. The DAI scores were lower in the DSS-induced mice that received TER administration vs that in the group treated with only DSS (Figure 1B). In addition, DSS typically caused colonic shortening, whereas such change was significantly improved in TER groups (Figure 1C). The severity of colonic inflammation was further evaluated by histopathological analysis (Figures 1D,E). The DSS group existed distortion of crypts, loss of goblet cells, and severe mucosal damage. However, administration of TER to DSS-induced UC mice could obviously improve the pathological changes in a dose-dependent manner. Only TER (20 mg/kg) did not generate a histopathological change in the colons of mice. Moreover, TER at 10 and 20 mg/kg significantly reduced the level of DSS-induced hyperactivated MPO (Figure 1F).

### TER Suppresses Pro-inflammatory Cytokines Secretion, NF-κB, and NLRP3 Inflammasome Activation in DSS-Treated C57BL/6 Mice

**Figure 2:**
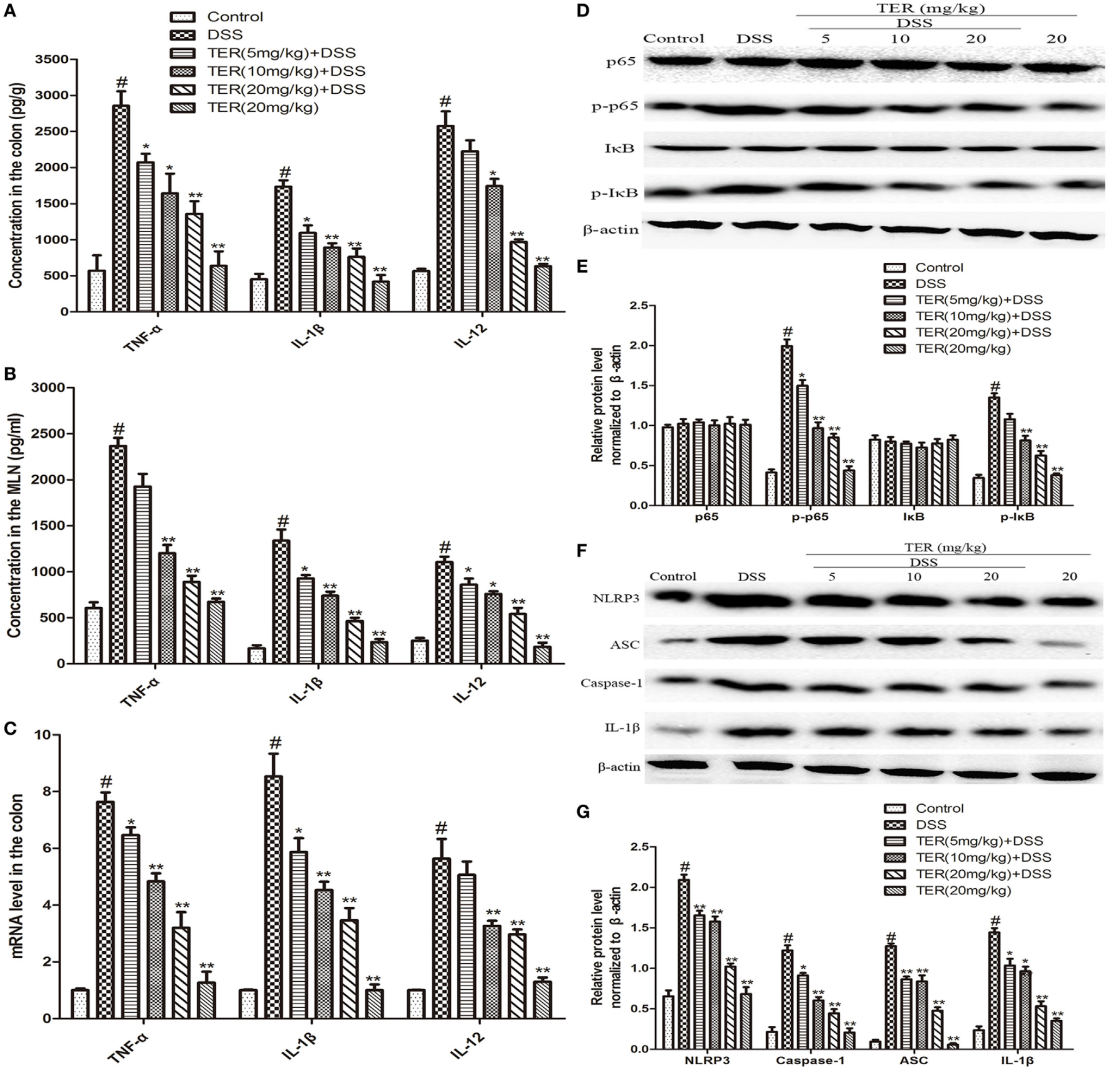
**Terpinen-4-ol (TER) reduces pro-inflammatory cytokines secretion and suppresses NF-κB and NLRP3 inflammasome activation in dextran sulfate sodium (DSS)-treated C57BL/6 Mice**. **(A)** The production of inflammation-related cytokines tumor necrosis factor α (TNF-α), interleukin-1β (IL-1β), and interleukin-12 (IL-12) in colonic cultures. **(B)** The production of inflammation-related cytokines TNF-α, IL-1β, and IL-12 in mesenteric lymph node stimulated with cecal bacterial lysates. **(C)** TNF-α, IL-1β, and IL-12 mRNA levels in the colon tissues were measured by quantitative real-time PCR. **(D,F)** Protein levels of NF-κB p65, IκB, NLRP3, ASC, caspase-1, and IL-1β in the colon tissues were analyzed by western blotting. **(E,G)** The relative protein expression of NF-κB p65, IκB, NLRP3, ASC, caspase-1, and IL-1β were normalized to β-actin. The results are representative of at least three independent experiments and expressed as mean ± SD. *p < 0.05 and **p < 0.01 vs the group treated with only DSS; ^#^*p* < 0.05 vs the control group.

The anti-inflammatory activity of TER was further confirmed by measuring TNF-α, IL-1β, and IL-12. The results showed that the levels of TNF-α, IL-1β, and IL-12 were significantly increased after DSS challenge. However, the increase of these cytokines was dramatically reduced by TER treatment in colon explants (Figure 2A). In addition, to investigate the effect of TER on host-dependent immune responses, we investigated the cytokine responses of the MLN cells to CBL. The results showed that TER could significantly inhibit the elevated expression of these cytokines in a dose-dependent manner (Figure 2B). In addition, we measured the mRNA levels of TNF-α, IL-1β, and IL-12 in colon tissues as well. Treatment with TER markedly decreased TNF-α, IL-1β, and IL-12 levels (Figure 2C).

NF-κB plays a pivotal role in regulating cytokines. To detect whether the suppression of inflammation by TER is mediated by NF-κB pathway, NF-κB p65 and IκB phosphorylation levels were determined. The resulted showed that the phosphorylation of p65 and IκBα was significantly increased in DSS group, but reversed in TER treatment groups (Figures 2D,E). Furthermore, emerging evidence suggests the pivotal role of NLRP3 inflammasome in the developments and pathogenesis of IBD (6). In our study, we have demonstrated that TER could decrease IL-1β production. Consequently, we further investigated whether TER could inhibit NLRP3 inflammasome activation in DSS-induced colitis mice. The results showed that the levels of NLRP3, ASC, Caspase-1, and IL-1β were remarkably enhanced after DSS challenge. The administration of TER (5, 10, and 20 mg/kg) inhibited the expression of production of NLRP3, ASC, Caspase-1, and IL-1β in a dose-dependent manner (Figures 2F,G).

### TER Inhibits Pro-inflammatory Cytokines Production, NF-κB, and NLRP3 Inflammasome Activation in LPS-Stimulated RAW264.7 Cells

**Figure 3:**
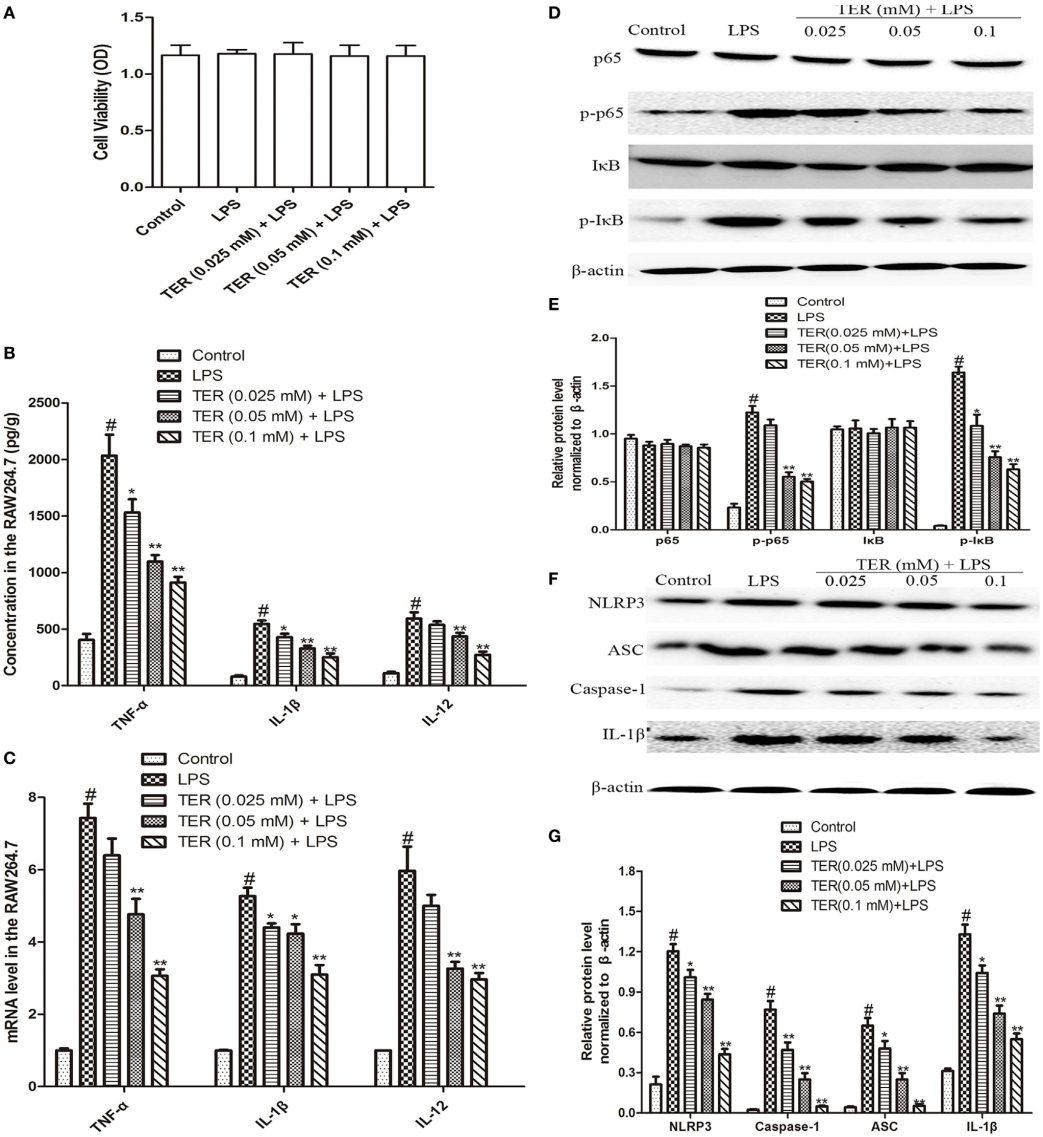
**Terpinen-4-ol (TER) decreases pro-inflammatory cytokines production and inhibits NF-κB and NLRP3 inflammasome activation in lipopolysaccharide (LPS)-stimulated RAW264.7 cells**. Cells were treated with different concentrations of TER (0.025–0.1 mM) in the absence or presence of LPS (1 µg/mL). **(A)** Cell viability was determined using MTT assay. **(B)** Tumor necrosis factor α (TNF-α), IL-1β, and interleukin-12 (IL-12) levels were detected by enzyme-linked immunosorbent assay. **(C)** TNF-α, interleukin-1β (IL-1β), and IL-12 mRNA levels were detected by quantitative real-time PCR. **(D,F)** Protein levels of NF-κB p65, IκB, NLRP3, ASC, caspase-1, and IL-1β in LPS-stimulated RAW264.7 cells were analyzed by western blotting. **(E,G)** The relative protein expression of NF-κB p65, IκB, NLRP3, ASC, caspase-1, and IL-1β were normalized to β-actin. The values presented are the means ± SD of three independent experiments. *p < 0.05 and **p < 0.01 vs the group treated with only LPS; ^#^*p* < 0.05 vs the control group.

Tumor necrosis factor-α, IL-1β, and IL-12 are three major cytokines in UC. As described above, TER suppressed the secretion of TNF-α, IL-1β, and IL-12 *in vivo* (Figures 2A–C). Subsequently, we confirmed the anti-inflammatory effect of TER *in vitro*. First, we detected the potential cytotoxicity of TER on RAW264.7 cells. The result showed that TER (0.025, 0.05, and 0.1 mM) had no toxic effect on RAW264.7 cells (Figure 3A). Thus, we detected the anti-inflammatory effects of TER on LPS-induced RAW264.7 cells with the concentration of 0.025, 0.05, and 0.1 mM. As expected, the expression of pro-inflammatory cytokines (TNF-α, IL-1β, and IL-12) was increased quickly in LPS-induced RAW264.7 cells. The concentration of TNF-α, IL-1β, and IL-12 was significantly reduced in TER treatment groups compared with LPS group (Figure 3B). To further test the effect of TER, mRNA expression levels of pro-inflammatory cytokines were evaluated by qRT-PCR. As shown in Figure 3C, gene expression levels of TNF-α, IL-1β, and IL-12 were also significantly increased by LPS stimulation compared with the control and clearly reduced by treatment with TER. These results demonstrated that TER inhibited the transcription and final secretion of TNF-α, IL-1β, and IL-12.

To test whether the anti-inflammatory effect of TER is mediated through NF-κB pathway, we measured the expression of NF-κB protein by western blotting. Our results showed that LPS significantly induced NF-κB activation in RAW264.7 cells. However, TER markedly inhibited LPS-induced phosphorylation of p65 and IκBα (Figures 3D,E). Furthermore, our *in vivo* study has also demonstrated that TER decreased IL-1β production (Figures 2A–C,F) and suppressed NLRP3 inflammasome activation (Figures 2F,G). To elucidate its anti-inflammatory mechanisms, we further explored whether TER could influence NLRP3 inflammasome activation *in vitro*. As shown in Figures 3F,G, TER significantly inhibited activated NLRP3 inflammasome-related protein levels and caspase-1 activity.

### NLRP3 Inflammasome Inhibition May Be Mainly Responsible for the Anti-inflammatory Effect of TER against DSS-Induced Colitis

**Figure 4:**
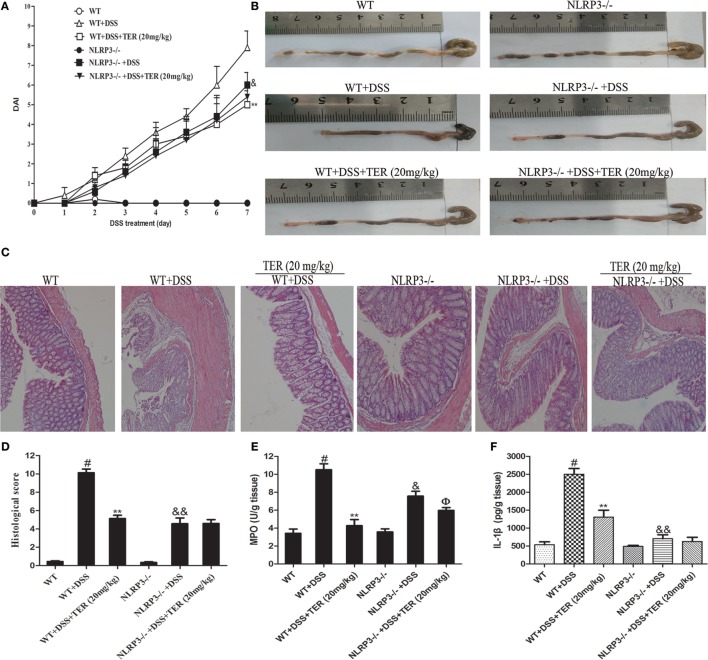
**NLRP3 deficiency protects mice from dextran sulfate sodium (DSS)-induced acute colitis**. Wild-type (WT) and NLRP3^−/−^ mice were treated with 2.5% DSS in their drinking water for 7 days to induce acute colitis. Twenty milligrams per kilogram terpinen-4-ol (TER) was administered for 14 days before and during DSS treatment *via* oral gavage once per day. Mice were sacrificed at day 21. **(A)** Disease activity index (DAI) during the disease process. **(B)** The lengths of colons from each group of mice were measured. **(C)** The colons from each experimental group were processed for histological evaluation (hematoxylin–eosin staining, 200×; scale bar, 50 µm). **(D)** Histopathological scores of each group were determined. **(E)** Myeloperoxidase (MPO) activity in the colon tissues was detected. **(F)** Protein level of interleukin-1β in colon tissues was determined by enzyme-linked immunosorbent assay. Data are presented as means ± SD (*n* = 4). **p < 0.01 vs the group treated with only DSS; ^#^*p* < 0.05 vs the control group. ^&^*p* < 0.05 and ^&&^*p* < 0.01 NLRP3^−/−^ vs WT mice in the group treated with only DSS. ^ɸ^*p* < 0.05 vs DSS-treated NLRP3^−/−^ mice group.

The DSS-induced mice colitis model was used to further elucidate the key role of NLRP3 inflammasome in TER protective effects by comparing WT mice and NLRP3^−/−^ mice. Consistent with the results from colitis experiment in C57BL/6 mice, administration of TER (20 mg/kg) showed a significant improvement on DSS-induced colitis of WT mice (Figures 4A–D). Results from Figures 4E,F also showed that DSS-increased MPO activity, and IL-1β level in the colon were significantly reduced by TER treatment in WT mice. However, we found that NLRP3^−/−^ mice were significantly protected from DSS-induced colitis, showing a less severity of symptoms, including reduced DAI scores and colon shortening compared with WT mice (Figures 4A–D). MPO activity and IL-1β level in colon of NLRP3^−/−^ mice also showed a significant inhibitory effect compared with those in the WT mice in response to DSS. Furthermore, histopathological analysis also revealed a significant decrease of mucosal damage, distortion of crypts, and loss of goblet cells. These results showed that mice deficient for NLRP3 were less sensitive to DSS-induced acute colitis. Consistent with the above results from colitis experiment in WT, TER (20 mg/kg) decreased the MPO activity in NLRP3^−/−^ mice (Figure 4E). However, we are surprising that TER did not protective effects on DSS-treated colitis in NLRP3^−/−^ mice characterized by DAI scores, colon shortening, pathological changes, and IL-1β secretion (Figures 4A–D,F). These results may suggest that NLRP3 inflammasome play an important role or at least play a partial role in TER-mediated colitis alleviation.

**Figure 5:**
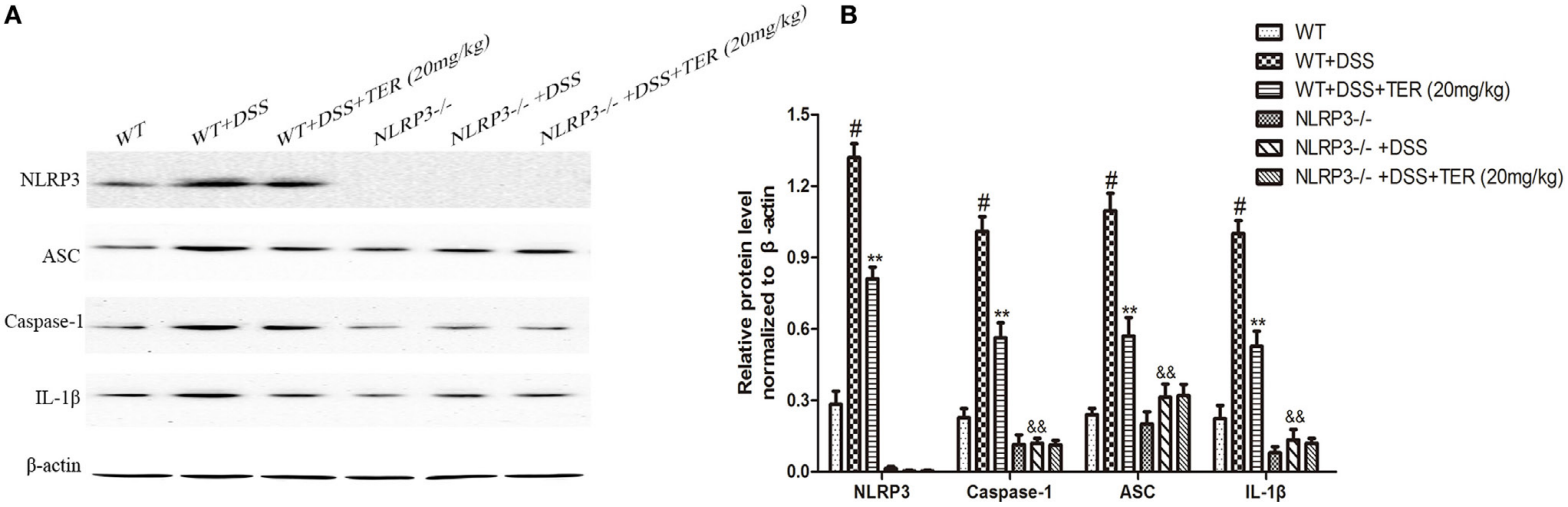
**NLRP3 inflammasome plays an important role in the development of dextran sulfate sodium (DSS)-induced acute colitis**. **(A)** Expressions of NLRP3, ASC, caspase-1, and interleukin-1β (IL-1β) in colon tissues were examined by western blotting. **(B)** The relative protein expressions of NLRP3, ASC, caspase-1, and IL-1β were normalized to β-actin. Data are presented as means ± SD (*n* = 3). *p < 0.05 and **p < 0.01 vs the group treated with only DSS; ^#^*p* < 0.05 vs the control group. ^&&^*p* < 0.01 NLRP3^−/−^ vs wild-type mice in the group treated with only DSS.

We further compared the activity of NLRP3 inflammasome in colon samples of WT and NLRP3^−/−^ mice by western blotting. As shown in Figures 5A,B, there is an undetectable level of NLRP3 protein in colon tissues of NLRP3^−/−^ mice. In addition, IL-1β level did not differ significantly between WT and NLRP3^−/−^ mice. However, high levels of caspase-1, ASC, and IL-1β were observed in the colon of WT mice receiving DSS, but not in those of NLRP3^−/−^ mice. Furthermore, DSS-increased caspase-1, ASC, and IL-1β levels in the colon were significantly decreased by TER treatment in WT mice, but did not in NLRP3^−/−^ mice. Our findings indicated that the NLRP3 inflammasome may play a critical role on DSS-induced colitis and its inhibition contributes to the anti-inflammatory effect of TER.

### TER Decreases Plasmatic LPS Concentrations and Optimizes the Number of *E. coli* and *Lactobacillus* in Mice Feces

**Figure 6:**
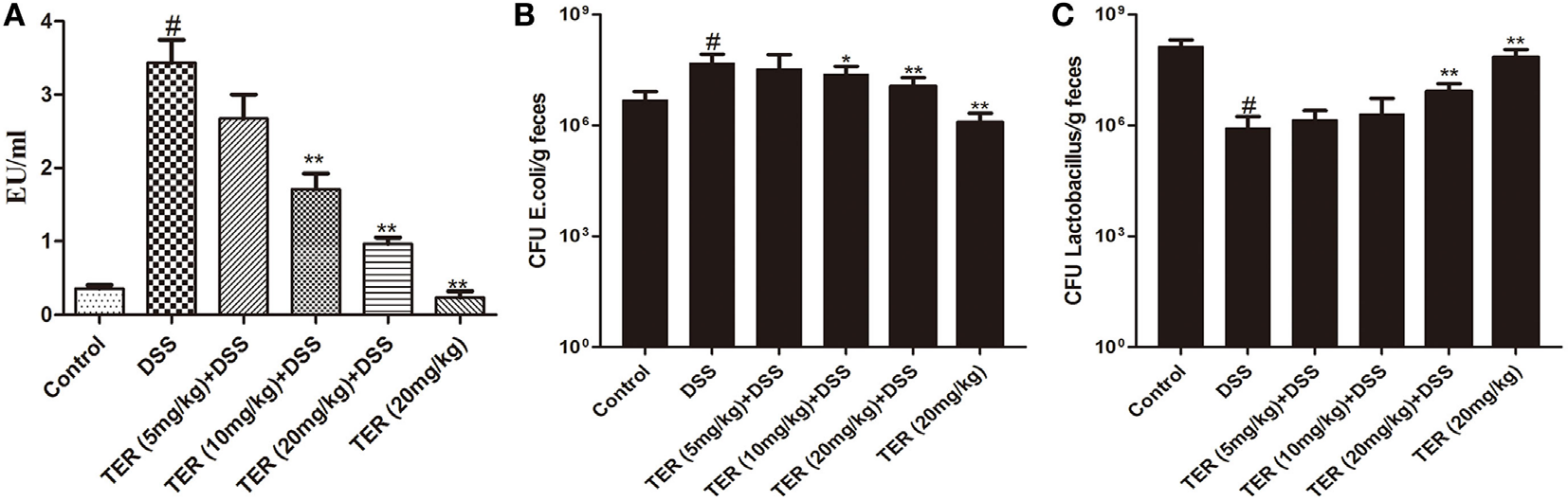
**Terpinen-4-ol (TER) decreases plasmatic lipopolysaccharide (LPS) concentrations and re-balance *E. coli* and *Lactobacillus***. **(A)** The plasmatic LPS concentrations were measured by Limulus amebocyte lysate assay. **(B)** *E. coli* and **(C)** *Lactobacillus* were detected by quantitative real-time PCR. Data are presented as means ± SD (*n* = 9). *p < 0.05 and **p < 0.01 vs the group treated with only dextran sulfate sodium (DSS); ^#^*p* < 0.05 vs control group.

To analyze whether TER could re-balance the composition of the commensal microbiota, representative bacteria in the feces of TER-treated mice were investigated by qRT-PCR. The copy numbers of *E. coli* were significantly reduced with TER treatments (10 and 20 mg/kg) compared with DSS group (Figure 6B). Furthermore, the levels of *Lactobacillus* were decreased by DSS compared with control group. However, TER (20 mg/kg) significantly increased the DSS-induced reduction of *Lactobacillus* levels (Figure 6C). LPS is a component of Gram-negative bacterial cells. We further detected the plasmatic LPS concentration as well. The result showed that plasmatic LPS concentration was significantly higher in DSS group than that in control group. However, the oral administration of TER suppressed plasmatic LPS level in a dose-dependent manner (Figure 6A).

### TER Contributes to the Maintenance of TJ Architecture *In Vivo* and *In Vitro*

**Figure 7:**
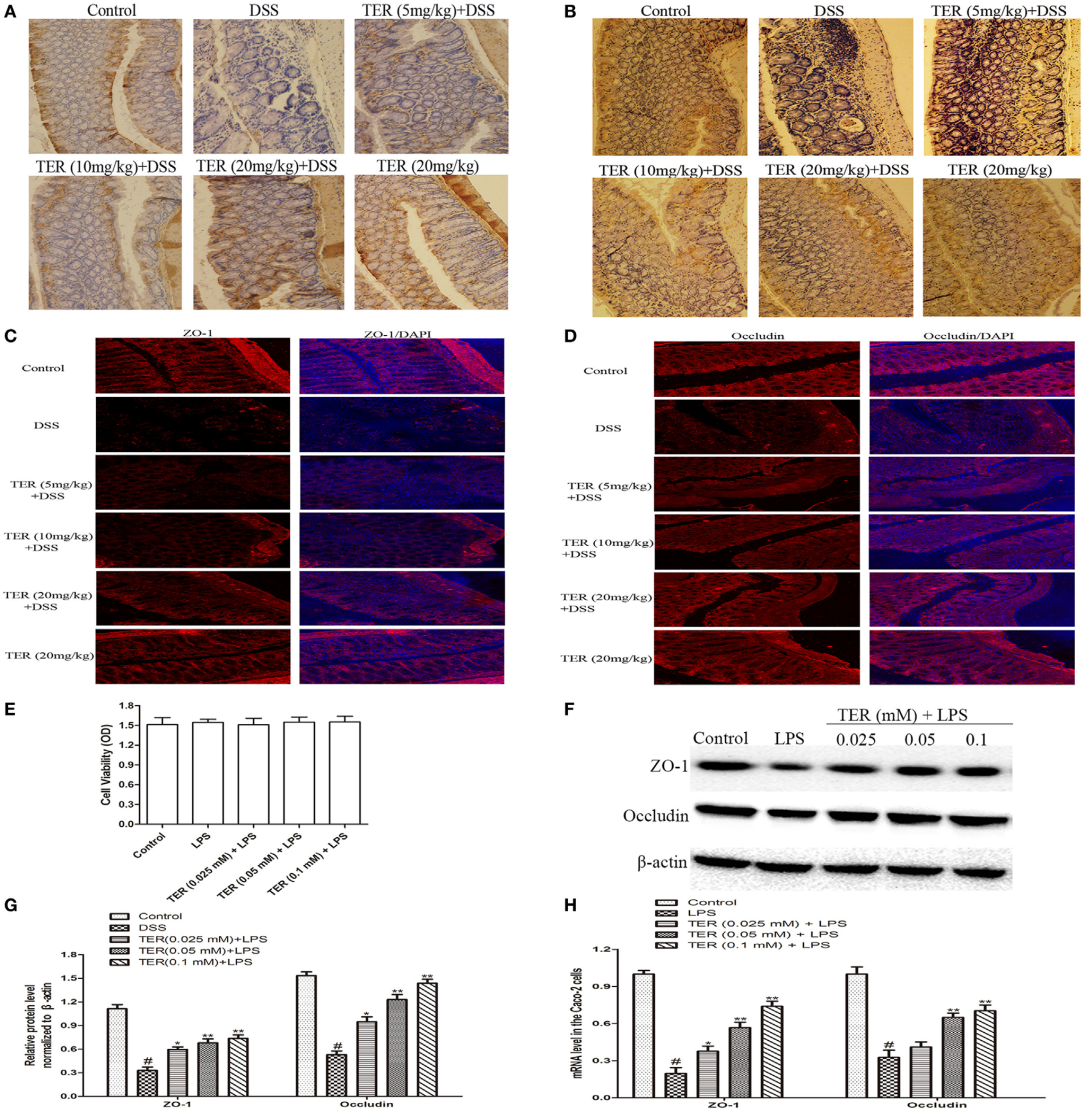
**Terpinen-4-ol (TER) contributes to the maintenance of tight junction architecture *in vivo* and *in vitro***. **(A)** Immunohistochemistry (IHC) for zonula occludens-1 (ZO-1) in colon tissue sections. Positive staining is brown colored. **(B)** IHC for occludin in colon tissue sections. Positive staining is brown colored. **(C)** Sections of colon tissue were immunostained with DAPI (blue) and anti-ZO-1 (red) and observed by confocal laser-scanning microscope, 200×; scale bar, 50 µm. **(D)** Sections of colon tissue were immunostained with DAPI (blue) and anti-occludin (red) and observed by confocal laser-scanning microscope, 200×; scale bar, 50 µm. **(E)** Caco-2 cells viability was determined using MTT assay. **(F)** ZO-1 and occludin protein levels were analyzed by western blotting. Cells were treated with different concentrations of TER (0.025, 0.05, and 0.1 mM) for 1 h in the absence or presence of lipopolysaccharide (LPS) (1 µg/mL). **(G)** The relative protein expression of ZO-1 and occludin were normalized to β-actin. **(H)** ZO-1 and occludin mRNA levels were analyzed by quantitative real-time PCR. The values presented are the means ± SD of three independent experiments. *p < 0.05 and **p < 0.01 vs the group treated with only LPS; ^#^*p* < 0.05 vs the control group.

Epithelial TJ proteins are an especially important aspect of the mechanical barrier, preventing harmful substances from breaching the mucosa, maintaining cellular integrity and permeability, and thus ensuring a relatively stable internal environment. ZO-1 and occludin are important epithelial TJ proteins. Thus, we detected the effect of TER on epithelial TJ proteins (ZO-1 and occludin). As shown in Figures 7A,B, ZO-1 and occludin positive signals were obviously decreased in the colon after DSS administration by immunohistochemical staining compared with the control group, while these changes were significantly increased through pretreatment with TER. IF analysis further demonstrated that ZO-1 and occludin were localized at the surface of the colonic epithelium in control group and TER (20 mg/kg) group. In DSS-treated mice, a substantial loss of staining intensity of ZO-1 and occludin was observed, which was significantly prevented by administration of TER (Figures 7C,D).

To extend our observations, we also detected the effect of TER on ZO-1 and occludin *in vitro*. First, we investigated the potential cytotoxicity of TER on Caco-2 cells. As shown in Figure 7E, TER (0.025, 0.05, and 0.1 mM) had no toxic effect on Caco-2 cells. We then detected the effect of TER on TJ proteins and mRNA expression. The results showed that the expression levels of ZO-1 and occludin were significantly downregulated in LPS-induced Caco-2 cells, and the TER-administered significantly increased their expressions in protein (Figures 7F,G) and mRNA (Figures 7H) levels compared with the LPS group, suggesting the importance of TER for restoring the integrity of the TJ networks.

## Discussion

Ulcerative colitis is a chronic and relapsing inflammatory disease of gastrointestinal tract, with high prevalence in developed countries (26). Genetic and environment, two initiative factors during inducing impaired intestinal tract barrier, could result in immune cell activation and cytokine production. However, the organism cannot eliminate acute mucosal inflammation by inhibiting inflammatory immune responses, and then inflammation develops. Currently, patients with UC are mainly treated with anti-inflammatory or immunosuppressive drugs, including glucocorticosteroids, immunosuppressive agents, and anti-TNF-α monoclonal antibody. However, most of these agents are inadequate and have severe side effects. TER, a kind of main components of essential oil from *Z. bungeanum* Maxim, has antioxidant, anti-inflammatory, and antimicrobial properties. However, direct evidence for the effects and mechanisms of TER on mice colitis has not yet been elucidated. In this study, we assessed the effects of TER on anti-UC and investigated the underlying mechanisms.

It is well known that DAI is the main parameters used to estimate the level of inflammation in UC (27). In this study, TER decreased DSS-induced DAI scores. As one indirect index, colon shortening outcomes were relieved in mice of TER groups. Meanwhile, it also markedly prevented DSS-induced destruction of the colonic tissues. Furthermore, TER decreased MPO activities. Hence, TER might be a promising candidate for the treatment of colitis.

Overproduction of pro-inflammatory cytokine production is a hallmark of colon damage in the development of UC (28). In our study, TER successfully alleviated acute colitis by suppressing the high-production of TNF-α, IL-1β, and IL-12 in colon explants. Moreover, the immune responses of the host are closely associated with UC. Therefore, we investigated the secretion of cytokines in MLN cells after stimulation with CBL. We found that the administration of TER also downregulated the production of inflammatory cytokines in MLN. NF-κB, a major inflammatory pathway, could regulate the production of cytokines. To test the inhibitory mechanism of cytokines production, we detected the effects of TER on NF-κB activation. As expected, TER inhibited DSS-induced NF-κB activation, resulting in alleviation cytokines secretions. Furthermore, the innate immune system is the first line to recognize microbes or endogenous molecules *via* pathogen-associated molecular patterns or damage-associated molecular patterns by host pattern recognition receptors. Inflammasome is a major component of innate immunity, and NLRP3 inflammasome in the inflammatory response plays the critical role and involves in diverse inflammatory diseases. Upon activation, NLRP3 proteins combine to apoptosis-associated speck-like protein adaptor (ASC) and subsequently induce the translocation and activation of pro-caspase-1, leading to the maturation and secretion of IL-1β (29). Unlike other cytokines, bioactive IL-1β production requires the activation of caspase-1 which converted pro-IL-1β into its mature active form relying on inflammasome activation (30–32). Furthermore, pharmacological inhibition of IL-1β was shown to successfully alleviate intestinal inflammation in colitis animal models (33). However, in the context of DSS colitis models, the role of the NLRP3 inflammasome in IBD is still much debatable. Allen et al. showed that NLRP3-deficient mice are more susceptible to develop colitis (34). On the other hand, Bauer et al. reported decreased sensitivity to DSS in mice lacking NLRP3. In the present study, we found that NLRP3 inflammasome was activated in DSS-induced colitis in C57BL/6 mice. And TER exerted its pharmacological effects through inhibiting the increase in protein levels of NLRP3, caspase-1, ASC, and IL-1β. Consistent with those obtained *in vivo*, in LPS-stimulated RAW264.7 cells, TER could inhibit the production of the cytokines during inflammation. In addition, we further found that TER suppressed NLRP3 inflammasome activated caspase-1 activity and subsequent IL-1β maturation and release. These data suggested that TER might protect against DSS-induced colitis by suppressing NLRP3 inflammasome activation.

To further verify the conclusion from our *in vivo* and *in vitro* study, we made more exploration in NLRP3^−/−^ mice. We found that oral administration of DSS in NLRP3^−/−^ mice induced a less severe colitis than WT mice and produced lower levels of MPO and IL-1β in colonic tissues, which suggested the important role of NLRP3 inflammasome in DSS-induced colitis. Meanwhile, TER did not significantly protect the development of DSS-induced colitis in NLRP3^−/−^ mice, suggesting that NLRP3 inflammasome may be involved in the preventive effect of TER on DSS-induced colitis. We compared the activity of NLRP3 inflammasome in colon samples of WT and NLRP3^−/−^ mice as well. We found that DSS administration increased the expression of caspase-1, ASC, and IL-1β in the colons of WT mice, but not in those of NLRP3^−/−^ mice. These findings indicated that the NLRP3 inflammasome inhibition may contribute to the anti-UC effect of TER.

Many studies have reported that intestinal barrier and intestinal microflora play key role in maintaining intestinal health (35, 36). The structural abnormalities in TJ proteins are the major cause of altered intestinal barrier in IBD patients (37). TJ proteins are capable of decreasing permeability of intestinal mucosa and restraining foreign substances across the intestinal mucosa, such as LPS, which results in inhibiting inflammation (38, 39). In addition, intestinal microflora is a critical player in intestinal permeability. An imbalance between beneficial and pathogenic bacteria is connected with IBD pathogenesis (40). *E. coli* and *Lactobacillus* are two representative bacteria and play important roles in normal and colitis mice. For example, *Lactobacillus*, a probiotics, could inhibit the growth of pathogenic bacteria and influence the adherence of pathogenic bacteria, such as *E. coli*, to the intestinal wall (41, 42). Indeed, several studies have also suggested that *Lactobacillus* level is decreased and *E. coli* level is increased in IBD patients (43). LPS is a component of Gram-negative bacterial cells, and its production is originated from intestinal microflora. Plasmatic LPS concentrations mainly relate to the balance of the intestinal microflora and intestinal barrier. In our study, administration of TER obviously inhibited the increase of plasmatic LPS induced by DSS. We then explored the intestinal bacteria and intestinal barrier. As expected, *E. coli* was significantly reduced by the TER treatments. Although the copy numbers of *Lactobacillus* did not be markedly changed in TER (5 mg/kg) and TER (10 mg/kg) groups, significantly increased in TER (20 mg/kg) group. Furthermore, TER might have a protective effect on barrier integrity by maintaining the expression of ZO-1 and occludin, thereby decreasing the severity of colitis.

In conclusion, our work explored a novel therapeutic strategy for UC. Administration of TER significantly attenuated DSS-induced colitis outcomes. The mechanisms of anti-UC involved two ways, the first one is associated with blocking of NLRP3 inflammasome; the second one is associated with the regulation of intestinal bacteria, colon epithelium barrier, and LPS production. Our results suggested that TER could potentially be used for the treatment of UC.

## Ethics Statement

All experimental protocols were guided in accordance with the approval of the Institutional Animal Care and Use Committee of our university under the approved protocol number SCXXK (JI-2016-0003).

## Author Contributions

Concept and design: ZZ, PS, YC, YF, BL, and NZ. Acquisition of data: ZZ, PS, JL, YL, and XL. Analysis and interpretation: ZZ, PS, BL, YC and NZ. Drafting and editing of the manuscript: ZZ, PS, XL, YL, BL, YC, and NZ. All the authors read and approved the final manuscript.

## Conflict of Interest Statement

The authors declare that the research was conducted in the absence of any commercial or financial relationships that could be construed as a potential conflict of interest.
